# Supplementary material for: Hemagglutinin Subtype Specificity and Mechanisms of Highly Pathogenic Avian Influenza Virus Genesis
Source: Viruses. 2022 Jul 19;14(7):1566. doi: 10.3390/v14071566 (PMC9321182; doi:10.3390/v14071566)
Supplement: Supplementary file 1 [file viruses-14-01566-s001.zip › Supplementary Materials Review HPAIV genesis.pdf]

Supplementary Table S1: Overview of AIV passaging experiments that focus on MBCS acquisition and virulence gain.

Viral strain (I/R[a])	Starting CS sequence	Methods	Results	CS changes[b]	Ref.
A/chicken/New Jersey/12508/1986 H5N2 (I)<br>A/chicken/Florida/27716-2 /1986 H5N2 (I)	PQKKKR (CCT CAG AAG AAA AAG AGA)	A) plaque selection in CEF[c] without trypsin (1 or 2 cycles).<br>B) 1 passage of trypsin-independent viruses from plaque selection in chickens.	A) plaque formation without trypsin.<br>B) decrease in MDT[d], increase in in vivo mortality.	No sequencing performed.	[213]
A/chicken/Pennsylvania/1/1983 H5N2 (I)	PQKKKR (CCT CAG AAG AAA AAG AGA)	9-10 passages on CEC[e] cells, alternately with and without trypsin and plaque selection (3 cycles).	Trypsin-independency.	HQRKKR (CAT CAG AGG AAA AAG AGA)<br>PKKKKR (CCT AAG AAG AAA AAG AGA)<br>PRKKKR (CCT CGG AAG AAA AAG AGA)<br>HQRKKR (CAT CAG AGG AAA AAG AGA)	[172]
A/turkey/Oregon/1971 H7N3 (I)	PENPKTR (CCG GAA AAT CCA AAG ACT AGA)	12 passages on CEC, alternately with and without trypsin.	Trypsin-independency and pathogenic for chickens.	Insertion of 24nts[f] at CS (28S rRNA).	[214]
A/turkey/Oregon/1971 H7N3 (I)	PENPKTR (CCG GAA AAT CCA AAG ACT AGA)	A) 7 passages in MDCK without trypsin and plaque purification (4 cycles).<br>B) 52 passages on CEC, alternately with and without trypsin. Plaque selection and more CEC passages.	A) trypsin-independency in MDCK, not in CEC. Not pathogenic in chickens.<br>B) trypsin-independency and pathogenic for chickens.	A) no changes.<br>B) 4 variants with insertion of 24nts at CS (28S rRNA). Variant 1 is described in [211]	[215]
A/seal/Mass/1/1980 H7N7 (I)	PENPKTR*G (CCA GAG AAT CCA AAG ACC AGA * GGA)	A) 7-10 passages in MDCK without trypsin.<br>B) 32-35 passages in CEC, alternately with and without trypsin.	A) trypsin-independency in MDCK, not in other cell types. Not pathogenic in chickens. B) modest trypsin-independency at p20. At p32 and p35 trypsin-independency and pathogenic for chickens.	A) no changes.<br>B) passage 32 PENPKTRG_RR* G_ (CCA GAG AAT CCA AAG ACC AGA GGA _CGG AGG * GGG_); passage 35 PENPKTRR_RR* G_ (CCA GAG AAT CCA AAG ACC AGA AGA _CGG AGG * GGG_)	[216]
A/chicken/Pennsylvania/21525/1983 H5N2 (I)	PQKKKR (CCT CAG AAG AAA AAG AGA)	Cloning in CEF and 2-4 passages in chickens.	Trypsin-independency and pathogenic for chickens.	No sequencing performed.	[204]
A/seal/Mass/1/1980 H7N7 (I)	PENPKTR*G (CCA GAG AAT CCA AAG ACC AGA * GGA)	Passaging in CEC (5x with thermolysin, 1x with trypsin, 1x without proteases).	Trypsin-independency.	Insertion of 60nts at CS (NP).	[164]

1) A/chicken/Pennsylvania/ 21525/1983 H5N2 (I)<br>2) A/turkey/Minnesota/1550 /1981 H5N2 (I)<br>3) A/environment/ Pennsylvania/937302/1993 H5N2 (I)<br>4) A/turkey/Ontario/7732/ 1966 H5N9 (R)	1) PQKKKR (AAG AAA AAG AGA)<br>2) RETR<br>3) RKTR (AGA AAA ACA AGA)<br>4a) PQRKKR (AGA AAA AAA AGA)<br>4b) RETR	3 passages of AF[g] in 10do[h] or 14do ECE.	1) in 14do ECE: trypsin-independency and pathogenic for chickens. 2) no changes.<br>3) in 14do ECE: trypsin-independency.<br>4a) in 14do ECE: trypsin-independency.<br>4b) no changes.	1) loss of GS p22[i].<br>2) no sequencing performed.<br>3) loss of GS p22.<br>4a) PQR*R*KKR (AGA *AGA* AAA AAA AGA)<br>4b) no sequencing performed.	[205]
A/emu/TX/39924/1993 H5N2 (I)	PQRKTR (AGA AAA ACA AGA)	1 passage in 14do ECE and 2 passages in chickens (spleen).	Trypsin-independency and pathogenic for chickens.	PQR*KR*KTR (AGA *AAA AGA* AAA ACA AGA)	[206]
A/whistling swan/Shimane/ 499/1983 H5N3 (I)	RETR (AGA GAA ACA AGA)	A) 18 passages in chicken air sacs.<br>B) 24 passages in chicken air sacs.<br>C) 24 passages in chicken air sacs and 3-5 passages in brain.	A) viral antigen present in endothelial cells of 3do chicks. No mortality upon IN[j] inoculation chickens.<br>B) more antigen present in 3do chicks. No mortality IN.<br>C) high mortality chickens IN.	A) RETR<br>B) REKR (AGA GAA AAA AGA)<br>C) R*R*KKR (AGA *AGA* AAA AAA AGA)	[196,207,208]
1) A/chicken/NewJersey /12508/1986 H5N2 (I)<br>2) A/chicken/Florida/27716 /1986 H5N2 (I)<br>3) A/chicken/Pennsylvania /21525/1983 H5N2 (I)	PQKKKR (CCT CAG AAG AAA AAG AGA)	A) cloning in CEF.<br>B) 1/2 passages in chickens.<br>C) cloning in CEF and passaging in chickens.<br>D) 1 passage in 14do ECE.	1/2A) unsuccessful.<br>1/2B) trypsin-independency and pathogenic for chickens.<br>3C) trypsin-independency and pathogenic for chickens. 3D) Trypsin-independency and pathogenic for chickens.	No sequencing performed.	[209]
A/chicken/Taiwan /K703-1/2008 H5N2 (I)	PQRKKR (CCC CAA AGG AAA AAA AGA)	4 passages in air sacs chicks and 4 passages in chickens.	Starting AIV was trypsin-independent, increased pathogenicity.	No changes.	[175]
1) A/chicken/Yokohama /aq-55/2001 H9N2 (R)<br>2) A/duck/Hokkaido/Vac-1 /2004 H5N1 (R)	1) PARKKR (CCT GCT AGA AAA AAG AGA)<br>2a) PRRKKR (CCT CGA AGA AAA AAA AGA)	Passaging in air sacs chicks.	1) trypsin-independent and moderately pathogenic.<br>2a) increased virulence at passage 3.<br>2b) increased virulence at passage 3.	1) loss of GS p22.<br>2a) PRR*R*KKR (CCT CGA AGA *AGA* AAA AAA AGA) at passage 1.<br>2b) no changes.	[173]

	2b) PQRERRKKR (CCT CAA AGA GAA AGA AGA AAA AAG AGA)				
A/Vietnam/1203/2004 H5N1 (R)	1) NSPQRERR-RKKR (aat agc cct caa aga gag aga aga aga aaa aag aga)<br>2) PQRETR (aat agc cct caa aga gag acg aga)<br>3) PQREKR (aat agc cct caa aga gag aag aga)<br>4) PQRKTR (aat agc cct caa aga aaa acg aga)<br>5) PQRKKR (aat agc cct caa aga aaa aag aga)<br>6) PQRERKKR (aat agc cct caa aga gag aga aaa aa gaga)<br>7) PQRRKKR (aat agc cct caa aga aga aaa aag aga)<br>8) PQRERRKKR (aat agc cct caa aga gag aga aga aaa aag aga)<br>9) PQRERRRKTR (aat agc cct caa aga gag aga aga aga aaa acg aga)<br>10) PQRERRQKKR (aat agc cct caa aga gag aga aga caa aaa aag aga)<br>11) NVPQRERRRKKR (aat gtc cct caa aga gag aga aga aga aaa aag aga)<br>12) RETR (aat agc cct caa aga gag acg aga)	1-11) ONO[k] inoculation of chickens, sequencing of brain/lung tissue with Sanger.<br>12/13) IV[l] inoculation of chickens, sequencing of brain.	1,2,6-9,11,12) Genetically stable<br>3-5,10,13) Genetically unstable	1,2,6-9,11,12) no changes.<br>3) NSLRREKR (aat agc ctt cga aga gag aag aga), NSHQREKR (aat agc cat caa aga gag aag aga)<br>4) NSPQRK*RK*TR (aat agc cct caa aga aaa *aga aaa* acg aga)<br>5) NSPQR*R*KKR (aat agc cct caa aga *aga* aaa aag aga)<br>10) RERRRKKR (aat agc cct caa aga gag aga aga cga aaa aag aga), RERRLKKR (aat agc cct caa aga gag aga aga cta aaa aag aga), RKRRQKKR (aat agc cct caa aga aag aga aga caa aaa aag aga)<br>13) NSPQRK*RK*TR (aat agc cct caa aga aaa *aga aaa* acg aga)	[165]

	13) RKTR (aat agc cct caa aga aaa acg aga)				
1) A/ostrich/South Africa /ORD/2012 H7N1 (I)<br>2) A/ostrich/South Africa /325863/2015 H5N2 (I)	1) PELPKGR (CCC GAA CTC CCA AAG GGA AGA)<br>2) PQRETR (CCT CAA AGA GAG ACA AGA)	Passaging in ECE (passage 1-7 AF, passage 8-17 AF and embryo).	Increased ECE mortality and decreased MDT.	No consensus changes, but very low frequencies of CS variants such as recombination with NP in virus (2).	[210]
A/chicken/England/11406/2008 H7N7 (R)	1) PEIPKKR (CCA AAG AAA AGA)<br>2) PEIPKGR (CCA AAG GGA AGA)	A) 10 passages of AF in 14do ECE.<br>B) 1 passage in 9do or 14do ECE, AIV selection in embryonic tissue.<br>C) 3 passages of embryonic tissue in 14do ECE.	A) no changes.<br>B/C) selection of variants with LP/HP mixed phenotype.	1A) no changes.<br>1B) PEIPKKKKR and PEIPKRKKR (no nucleotide sequences available).<br>2B) no changes. 2C) no changes.	[211]
A/whistling swan/Shimane/499/1983 H5N3 (R)	1) PQRKKR (AGA AAA AAA AGA)<br>2) PQRKKR (CGC AAG AAG AGA)	A) single passage in MDCK without trypsin.<br>B) intracerebral passage in chicks.	1A) plaque formation without trypsin.<br>2A) no plaque formation.<br>1B) increased mortality.<br>2B) no changes.	1A) R*KKR*KKR (AGA *AAA AAA AGA* AAA AAA AGA), R*R*KKR (AGA *AGA* AAA AAA AGA)<br>1B) R*KKR*KKR (AGA *AAA AAA AGA* AAA AAA AGA).<br>2A/B) no changes.	[212]

[a] I/R: the stock used for the passaging experiments was an isolate (I) or recombinant virus produced through reverse genetics (R).

[b] Inserted nucleotides have been indicated in italics.

[c] CEF: chicken embryonic fibroblasts.

[d] MDT: mean death time.

[e] CEC: chicken embryonic cells.

[f] nts: nucleotides.

[g] AF: allantoic fluid.

[h] ndo ECE: n-day-old embryonated chicken eggs.

[i] GS p22: putative glycosylation site p22 (H3 numbering).

[j] IN: intranasal.

[k] ONO: oronasal-ocular.

[l] IV: intravenous.

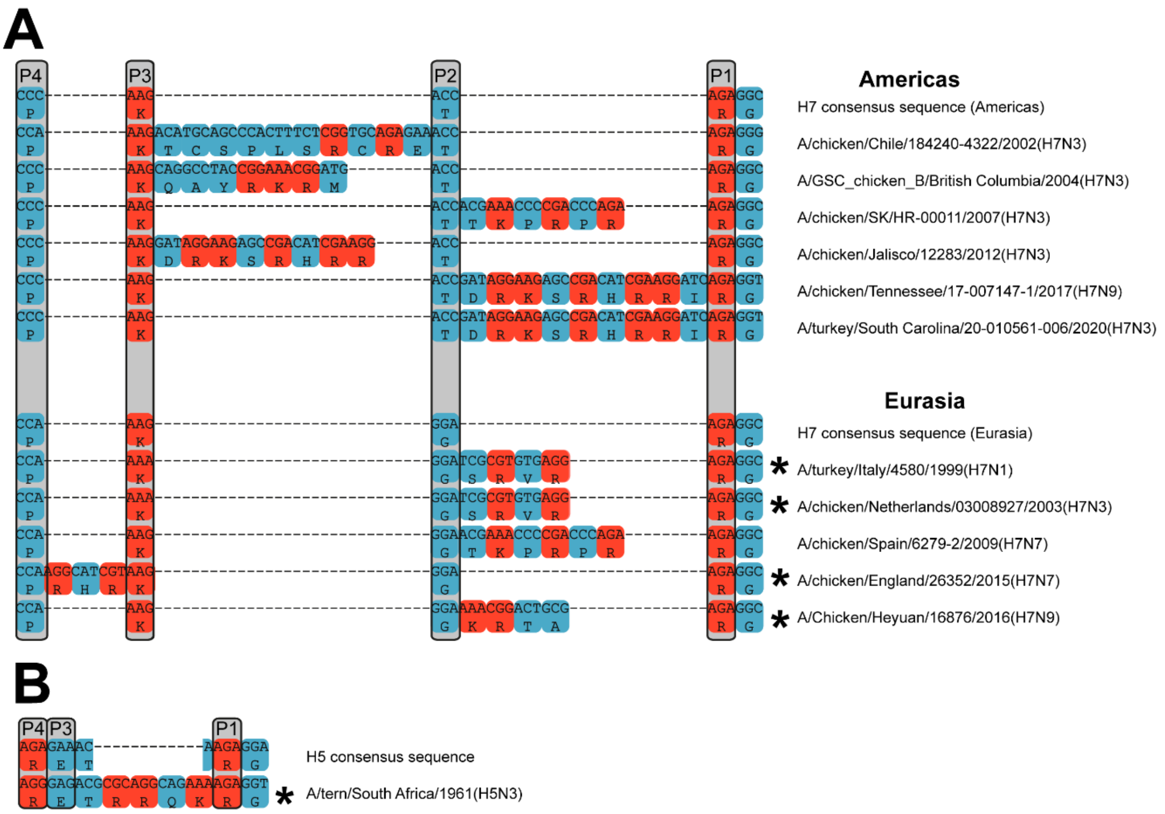

Supplementary Figure S1: Alternative alignments of MBCS-containing H5 and H7 AIVs that propose NHR as the mechanism of MBCS acquisition. Nucleotide and amino acid alignments of H7 (A) and H5 (B) MBCS-containing AIVs that are considered to be confirmed or putative (indicated by an asterisk) cases of NHR. The grey boxes delineate conserved amino acids from the LPAI consensus sequence with P1 to P4 indicated on top. Arginines and lysines are depicted in red and all other amino acids are depicted in blue.
